# Monoaminergic and Histaminergic Strategies and Treatments in Brain Diseases

**DOI:** 10.3389/fnins.2016.00541

**Published:** 2016-11-24

**Authors:** Giuseppe Di Giovanni, Dubravka Svob Strac, Montse Sole, Mercedes Unzeta, Keith F. Tipton, Dorotea Mück-Šeler, Irene Bolea, Laura Della Corte, Matea Nikolac Perkovic, Nela Pivac, Ilse J. Smolders, Anna Stasiak, Wieslawa A. Fogel, Philippe De Deurwaerdère

**Affiliations:** ^1^Department of Physiology and Biochemistry, University of MaltaMsida, Malta; ^2^Division of Molecular Medicine, Rudjer Boskovic InstituteZagreb, Croatia; ^3^Departament de Bioquímica i Biologia Molecular, Facultat de Medicina, Institut de Neurociències, Universitat Autònoma de BarcelonaBarcelona, Spain; ^4^School of Biochemistry and Immunology, Trinity College DublinDublin, Ireland; ^5^Department of Neuroscience, University of FlorenceFlorence, Italy; ^6^Department of Pharmaceutical Chemistry and Drug Analysis, Vrije Universiteit BrusselBrussels, Belgium; ^7^Department of Hormone Biochemistry, Medical University of LodzLodz, Poland; ^8^Centre National de la Recherche Scientifique (Unité Mixte de Recherche 5293), Institut of Neurodegenerative DiseasesBordeaux Cedex, France

**Keywords:** antipsychotic, antidepressant, monoamine oxidase inhibitor, multi-target pharmacology, neurodegenerative diseases, stroke, antiparkinsonian treatments, drug addiction

## Abstract

The monoaminergic systems are the target of several drugs for the treatment of mood, motor and cognitive disorders as well as neurological conditions. In most cases, advances have occurred through serendipity, except for Parkinson's disease where the pathophysiology led almost immediately to the introduction of dopamine restoring agents. Extensive neuropharmacological studies first showed that the primary target of antipsychotics, antidepressants, and anxiolytic drugs were specific components of the monoaminergic systems. Later, some dramatic side effects associated with older medicines were shown to disappear with new chemical compounds targeting the origin of the therapeutic benefit more specifically. The increased knowledge regarding the function and interaction of the monoaminergic systems in the brain resulting from *in vivo* neurochemical and neurophysiological studies indicated new monoaminergic targets that could achieve the efficacy of the older medicines with fewer side-effects. Yet, this accumulated knowledge regarding monoamines did not produce valuable strategies for diseases where no monoaminergic drug has been shown to be effective. Here, we emphasize the new therapeutic and monoaminergic-based strategies for the treatment of psychiatric diseases. We will consider three main groups of diseases, based on the evidence of monoamines involvement (schizophrenia, depression, obesity), the identification of monoamines in the diseases processes (Parkinson's disease, addiction) and the prospect of the involvement of monoaminergic mechanisms (epilepsy, Alzheimer's disease, stroke). In most cases, the clinically available monoaminergic drugs induce widespread modifications of amine tone or excitability through neurobiological networks and exemplify the overlap between therapeutic approaches to psychiatric and neurological conditions. More recent developments that have resulted in improved drug specificity and responses will be discussed in this review.

## Introduction

Monoaminergic systems are important cellular targets in a variety of neuropsychiatric and neurological conditions. Improved knowledge of these systems, in terms of function and molecular organization, has confirmed their roles in the efficacy of older medications. Medicinal chemistry has detected some newer compounds that affect identified molecular targets and additional monoaminergic targets are still being investigated. Although monoaminergic systems have a pivotal role in the effect of many existing medicines, there is a need for further developments, as additional targets have been identified that could be as interesting as monoamines (Millan et al., 2015b).

Monoaminergic systems, involving dopamine (DA), serotonin (5-HT), noradrenaline (NA) and histamine are involved in virtually all cerebral functions and modification of their activity has been identified in most, if not all, neuropsychiatric and neurological diseases. Some of these diseases, including schizophrenia, depression and Parkinson's disease (PD) benefit from monoaminergic-based treatments, but there is still scope for therapeutic improvement. In spite of clear monoaminergic disturbances in other devastating diseases, including Alzheimer's disease, addiction and epilepsy, no approved monoamine-based treatments are yet available. The discovery of the most pertinent drugs in numerous psychiatric diseases was serendipitous while the causal implication of monoaminergic systems in several diseases is still unclear. In this context, the identification of drug targets as well as more detailed neuropharmacological analysis should allow for the improvement of existing drugs.

The complexity of the organization of monoaminergic systems in the brain limits the likelihood of drugs acting on a single target to correct a disease selectively, although recent examples suggest that this strategy might be worth continuing (Meltzer and Roth, 2013). Moreover, specific targets for a neurotransmitter are often distributed in numerous neurobiological circuits so that a selective compound may produce a plethora of distinct effects, some of which may be undesirable. Such a situation may occur in the treatment of diseases like schizophrenia where the use of DA receptor antagonists is associated with undesirable side effects, as discussed below. The complexity of these circuits may lead to the proposed use of either agonists or antagonists for correcting the same diseases (Di Giovanni and De Deurwaerdère, 2016). There is a growing body of evidence demonstrating that most neuropsychiatric disorders are multifactorial and that an action on a single target is elusive both in terms of symptomatic and curative treatments (Millan et al., 2015a,b). Increased understanding of the mechanisms involved in such diseases led to the use of combinations of drugs targeting distinct receptors/enzymes or to the development of multi-target drugs. Finally, it is clear that the action on one target of one monoaminergic system will have repercussion on the activity of the other systems, which can affect the efficacy of medicines and may also introduce unwanted side-effects.

**Figure 1:**
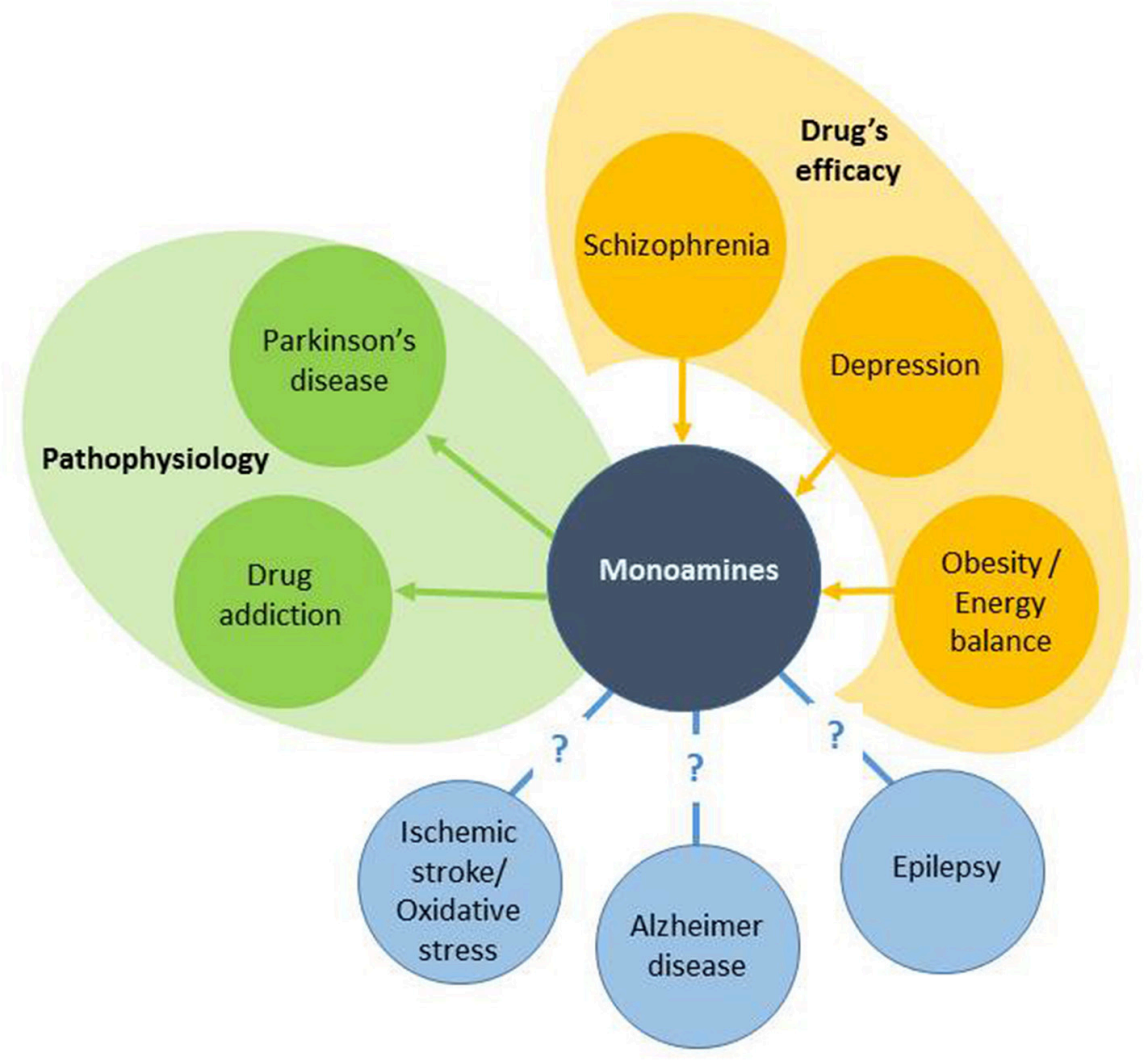
**Relationships between brain diseases and monoamines**. We artificially separate three groups of diseases. The first group is based on the discovery of the involvement of monoamines in drug's efficacy (the arrows go from the drugs to monoamines in a disease). The second group includes diseases where monoamines have been causally involved in the disease, leading to development of monoaminergic drugs (L-DOPA for instance; the arrows go from the disease to the drugs). The third group has no monoamine-based treatments implying an open research.

Therapeutic approaches that target monoaminergic systems are completely different and depend on the disease. The aim of this review is to present the development of new chemical compounds and therapeutic strategies aimed at modulating monoaminergic function in various brain disorders. We used the angle depicted in Figure 1 arbitrarily categorizing three groups of diseases. Thus, after briefly presenting the physiology of the four main monoamine systems in the brain, this review will summarize past and current developments in the treatment of neuropsychiatric diseases that have monoaminergic-based medication (schizophrenia, depression, obesity) without a clear pathophysiological picture. Thereafter, we will present therapeutic strategies for brain diseases where the cause of the disease has been identified but with distinct success regarding the treatments and their amelioration (PD, drug addiction). The review will finish by presenting some strategies aimed at ameliorating the symptoms of brain diseases that still do not benefit from monoaminergic-based treatments in spite of the numerous evidence that it could be beneficial (epilepsy, AD, and ischemic stroke). The choice has been also driven by the involvement of the various researchers in the COST action CM1103, which was dedicated to drug design in the field of monoamines. The overall picture should give a clearer indication of the neuropharmacological significance of targeting monoaminergic systems in neuropsychiatric diseases and the need for future research.

## Monoaminergic systems in the brain

**Figure 2:**
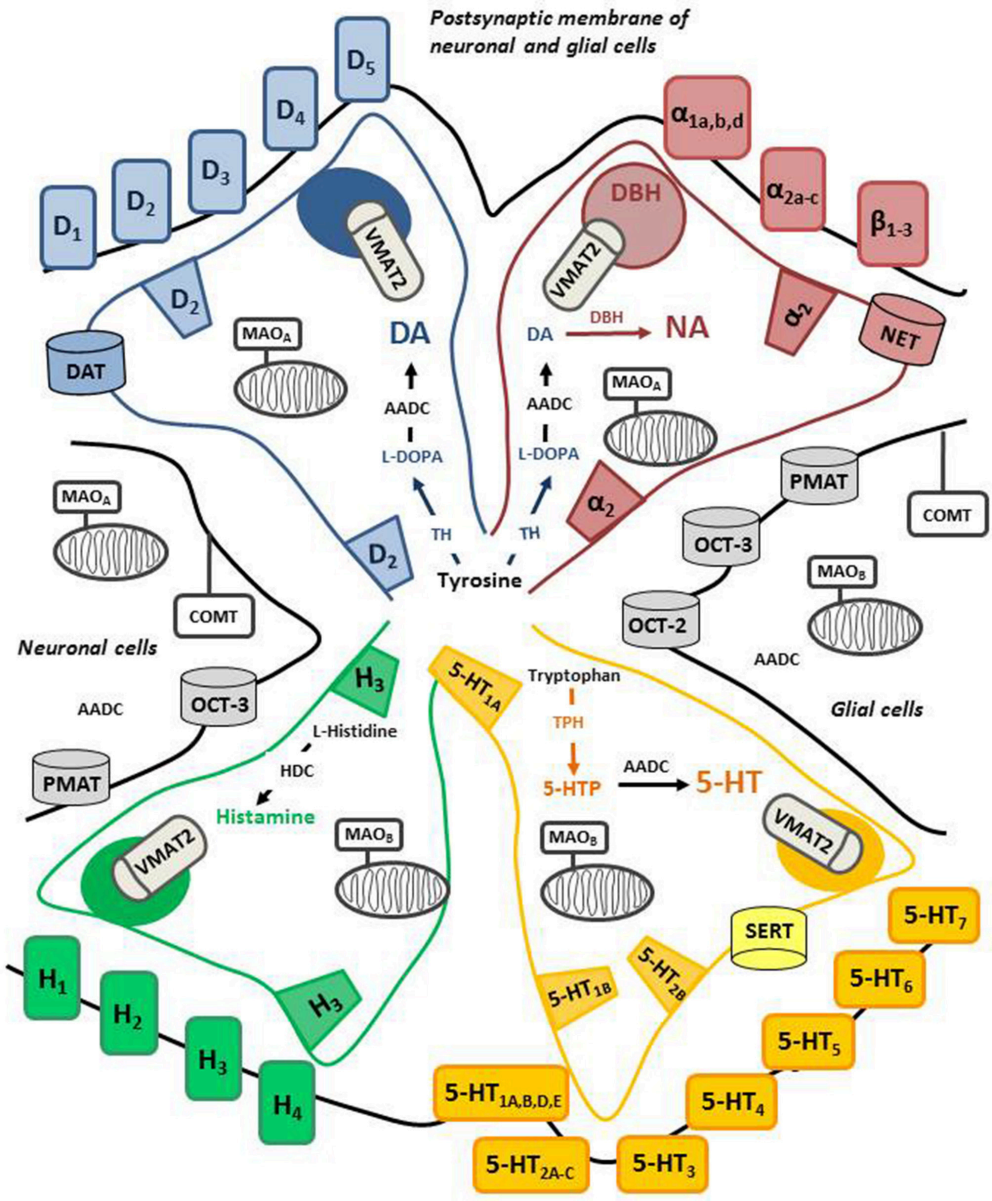
**Cellular and molecular organization of central monoaminergic systems**. The figure depicts each monoamine system (dopamine, DA; noradrenaline, NA; serotonin, 5-HT; histamine) the biosynthesis, metabolism, the receptors and transporters. The color is used to identify the proteins that are selective for each system while the black color is used for non-specific proteins. The terminals of each monoaminergic neurons contact post-synaptic elements that express a variety of receptors which are more or less specific for each monoamine. Autoreceptors can be located at terminals and cell bodies for most systems. In the case of serotonergic cells, 5-HT_1A_ autoreceptors are expressed at cell bodies and 5-HT_1B_ autoreceptors are expressed at terminals The post-synaptic elements (neurons, glial cells) also express enzymes involved in their metabolism (MAO-A/B, COMT, AADC) as well as non-specific transporters. Of note, the distribution of MAO-B in the serotoninergic cells is rather located at the level of cell bodies. DBH is mainly expressed in vesicles of exocytosis in noradrenergic terminals. AADC, aromatic L-amino acid decarboxylase; DBH, dopamine β-hydroxylase; TPH, tryptophan hydroxylase; VMAT2, vesicular monoamine transporter; SERT, 5-HT transporter, DAT, DA transporter; NET, NA transporter; OCT, organic cation transporters; PMAT, plasma membrane monoamine transporter; HDC, L-histidine decarboxylase; MAO, monoamine oxidase (A or B); COMT, catechol-O-methyl transferase.

Pathways of monoaminergic systems project from the brainstem to different brain areas and the spinal cord, and they include dopaminergic neurons, located in the ventral tegmental area (VTA) and the substantia nigra pars compacta (SNc), noradrenergic neurons, arising from locus coeruleus (LC), serotonergic neurons, originating from median and dorsal raphe nuclei (Hale and Lowry, 2011) and histaminergic neurons arriving from tuberomamillary nucleus of the hypothalamus (Taylor, 1975). Figure 2 illustrates approximately the, still limited, knowledge of the pharmacological and biochemical identities for each of these systems. DA is a catecholamine synthesized by the enzyme tyrosine hydroxylase (TH), which catalyzes the conversion of the amino acid L-tyrosine to L-3,4-dihydroxyphenylalanine (L-DOPA), and by the aromatic L-amino acid decarboxylase (AADC) which converts L-DOPA into DA (Cooper et al., 2003). DA can be metabolized by dopamine β-hydroxylase (DBH) into NA. DBH is mainly present in vesicles and can be found in the cytosol (Gagnon et al., 1976). 5-HT is produced from L-tryptophan by tryptophan hydroxylase (TPH) and AADC. Unlike in the case of the catecholamines and 5-HT, the decarboxylase involved in the synthesis of histamine is L-histidine decarboxylase (Taylor, 1975).

The degradation of biogenic amines is a complex mechanism that is not fully understood because it involves several enzymes and cell types. The monoamine oxidases (MAO) are a family of flavin adenine dinucleotide (FAD)-containing enzymes that catalyze the oxidative deamination of primary, secondary or tertiary amines. They produce hydrogen peroxide, ammonia and the corresponding aldehyde. There are two isoforms of MAO present in mammals: MAO-A and MAO-B, which share approximately 70% sequence identity and are encoded by separate genes located on the X chromosome (Youdim et al., 2006). The anatomical distribution of MAO isoforms in human brains was confirmed by positron-emission topography (PET) using intravenous ^11^C-labeled irreversible inhibitors (Fowler et al., 1987). Autoradiographic and *in situ* hybridization studies have revealed that histaminergic and serotonergic neurons as well as astrocytes are rich in MAO-B whereas catecholaminergic neurons mainly contain MAO-A (Saura et al., 1992, 1996). DA, NA and 5-HT can be degraded by MAO-A (Youdim et al., 2006; Finberg, 2014). The aldehyde derivatives, which are more toxic than the parent compounds, are catabolized by aldehyde dehydrogenase/reductase (Eisenhofer et al., 2004). MAO-B is involved in the glial metabolism of catecholamines and can affect the metabolism of biogenic amines in diverse conditions including PD (Youdim et al., 2006; Riederer and Laux, 2011). It is also involved in the metabolism of other amines, including 2-phenylethylamine and N(tele)-methylhistamine.

Catechol-O-methyltransferase (COMT) can also metabolize DA and NA (Eisenhofer et al., 2004). Two isoforms have been described (soluble COMT, S-COMT; membrane-bound COMT, MB-COMT) having different subcellular compartmentation. In the brain, S-COMT is found in the cytosol of glial cells, whereas MB-COMT is bound to the endoplasmic reticulum and present in neurons. MB-COMT mainly inactivates catecholamines and derivatives originating from DA and NA neurotransmission, whereas S-COMT preferentially transforms exogenous catecholamines (Myohanen et al., 2010; Tammimäki et al., 2010). COMT is not present in DA terminals (Schendzielorz et al., 2013), implying that glial cells or other neurons reuptake extracellular DA in tissues where the clearance of DA is low. COMT is also involved in the degradation of metabolites generated by MAO activities. The final products from these two distinct catabolic pathways is homovanillic acid for DA metabolism and either vanillylmandelic acid or a conjugated form of 3-methoxy-4-hydroxyphenylglycol for NA metabolism (Eisenhofer et al., 2004). Histamine follows distinct pathways. It is converted in the brain by histamine N-methyltransferase to N(tele)-methylhistamine, which undergoes degradation by MAO-B followed by the aldehyde oxidation to N(tele)-methylimidazole acetic acid, while in periphery histamine is degraded by diamine oxidase (Taylor, 1975; Morisset et al., 2000).

Once synthesized, all monoamines are concentrated in the vesicular compartment by the vesicular monoamine transporter or VMAT2 (Wimalasena, 2011). Regarding the extracellular space, various transporters are involved in the clearance of monoamines. Three major classes of monoamine transporters, 5-HT transporter (SERT), DA transporter (DAT), and NA transporter (NET), are responsible for the reuptake of 5-HT, DA and NA, respectively, from the synaptic cleft back to pre-synaptic neuron (Torres et al., 2003). Of note, the reuptake of DA occurs physiologically through the NET in extrastriatal tissues (Di Chiara et al., 1992). The reuptake of these monoamines is not totally selective and there are also several low affinity/high capacitance transporters including the organic cation transporters (OCT1-3 subtypes) and the plasma membrane monoamine transporter (PMAT) (Daws, 2009; Hensler et al., 2013; De Deurwaerdère et al., 2016). These systems, which are expressed by glial cells and other types of neurons, are involved in the reuptake of histamine (no preferential transporter) and the other monoamines. Thus, while the first step of synthesis can be selective, most of the other steps are overlapping and the catabolism involves cells other than monoaminergic neurons (Figure 2). Furthermore, the enzymes of biogenic amine catabolism are involved in several additional processes.

The main selectivity regarding these systems, although limited to some extent, is conferred by the receptors; two families for DA (D_1_-like including D_1_ and D_5_R and D_2_-like including D_2_L, D_2_S, D_3_, D_4_ receptor subtypes) (Emilien et al., 1999; Beaulieu and Gainetdinov, 2011), three families also for NA (α_1a,b,d_ or α_2a−d_ receptor subtypes, and β receptor subtypes including β_1−3_) (Andersson, 1980; Ahles and Engelhardt, 2014; Ghanemi and Hu, 2015), four for histamine (H_1−4_) (Panula et al., 2015) and seven for 5-HT (5-HT_1−7_) (Barnes and Sharp, 1999; Hoyer et al., 2002). They are mostly G-protein-coupled receptors (GPCR), except for the 5-HT_3_ receptor subtypes which are channel receptors. The cerebral distribution is distinct for each receptor. The differential distribution in some brain regions for a subtype can be a hint with regards to its participation in some specific functions. Details related to brain expression of monoamine receptors are found in the above-cited authoritative reviews. In any case, most of them are expressed in several neurobiological networks. Moreover, several subtypes may directly, as autoreceptors (see below), or indirectly modify the activity of monoaminergic neurons, thereby altering neurobiological networks beyond their specific distribution.

Each monoaminergic neuronal system has its own autoreceptor(s). Thus, adrenergic cells express the α_2c_ receptor while histaminergic neurons express H_3_ receptors. Dopaminergic and serotonergic neurons express D_2_/D_3_ receptors and 5-HT_1A_, 5-HT_1B/1D_ and 5-HT_2B_ receptors, respectively. The function of these autoreceptors located at both cell bodies and/or neuron terminals is usually to inhibit the electrical activity, excitability, synthesis and/or release. They also regulate the activity of the transporters presumably via intracellular signaling pathways. As illustrated in Figure 2, these autoreceptors are also expressed by other cell types (heteroreceptors).

The last point to consider when dealing with neuropharmacology of monoaminergic systems is that, either by acting on non-selective enzymes such as MAO, or by selectively targeting one receptor, the biological responses likely involve all systems due to their interaction throughout the brain (Fitoussi et al., 2013; Hensler et al., 2013). The complex interaction between these systems overwhelms the single target and the integrative views of the newer monoaminergic treatments must clearly include this dimension (De Deurwaerdère and Di Giovanni, 2016).

## From the drugs to monoamines

### Treatments of schizophrenia

#### Historical perspectives

It is logical to start the overview of the monoaminergic potentials in psychiatry by the discovery of neuroleptic drugs (antipsychotic drugs), which may be considered as the birth of biological psychiatry. Schizophrenia is a complex neuropsychiatric disorder with high prevalence (Owen et al., 2016). In the early 1950s, chlorpromazine was initially used by the French physicians Laborit and Charpentier for its antihistaminic properties, to calm down patients before anesthesia. The effect of chlorpromazine was spectacular and it was transposed successfully to the treatment of schizophrenia by Deniker and Delay (López-Muñoz et al., 2005). Other drugs from different chemical classes were found to mimic the antipsychotic action of chlorpromazine, but it was several years before the understanding that dopaminergic system could be involved (Carlsson and Lindqvist, 1963), and that the DA (D_2_) receptor subtype was a common target of all antipsychotic drugs (Seeman et al., 1975, 1976). Nowadays, central D_2_ receptors, among other receptor types, remain an essential target in drug design of newer antipsychotic drugs (Butini et al., 2016).

#### Mechanisms of action of antipsychotic drugs

All antipsychotic drugs block the D_2_ receptor subtype and the antipsychotic efficacy depends on the proportion of occupied D_2_ receptors, which is directly related to the affinity of the compound at D_2_ receptor and the free plasma concentration of the antipsychotic drug. Most of these drugs including chlorpromazine or haloperidol are efficacious against positive symptoms of schizophrenia such as delusion, paranoia or hallucinations. Their therapeutic benefit is assumed to be related to the blockade of D_2_ receptors in the ventral striatum (nucleus accumbens). On the other hand, these compounds are not efficacious against the negative symptoms which may even aggravate (Millan et al., 2014; Remington et al., 2016). These drugs also induce several serious side-effects, either due to their off-targets including H_1_, α_2_, muscarinic2 (M_2_) receptor antagonist properties, or to chronic central D_2_ receptor blockade. Notably the extrapyramidal side-effects (EPS) include parkinsonism, akathisia, dystonia and tardive dyskinesia (Ebadi and Srinivasan, 1995).

Although clozapine is more efficacious against negative and cognitive symptoms than other antipsychotic drugs and has fewer EPS (Ashby and Wang, 1996), its clinical use was discontinued due to its propensity to induce agranulocytosis. However, it was subsequently reintroduced if carefully monitored (Deutch et al., 1991; Ashby and Wang, 1996). The peculiar clinical responses gave rise to the concept of the typical and atypical antipsychotic drugs, the latter inducing less EPS and vegetative effects (Meltzer, 1993, 1999). Studies of the pharmacological behavior of drugs like clozapine or risperidone led to the proposal that the atypical profile corresponds to a higher affinity of the drug toward 5-HT_2_ (i.e., 5-HT_2A_) vs. D_2_ receptor subtypes (Meltzer et al., 1989a,b). This pharmacological constraint has been added to the D_2_ receptor target in the selection of drugs possibly exhibiting an atypical antipsychotic profile. Drugs like olanzapine, quetiapine, ziprasidone and more recently brexpiprazole are representatives of this class (Table 1). These newer antipsychotic drugs have lower propensity to induce EPS, but another concern emerges regarding the development of metabolic side effects such as weight excess. It is possible that their antagonist/inverse agonist profile at 5-HT_2C_ receptors in addition to the H_1_ receptor antagonism participate to the development of increased body weight and obesity side effect (see below) (Table 1).

**Table 1:** **Receptor-binding affinities of typical and atypical antipsychotic drugs**.

**Antipsychotics**	**D_2_**	**D_3_**	**5-HT_1A_**	**5-HT_2A_**	Other targets^*^
**TYPICAL ANTIPSYCHOTICS**					
Haloperidol	8.84	8.56	6.29	7.28	D_4_ (8.83); α_1A_ (7.9); σ_1_ (8.52)
flupentixol	9.46	8.76	5.1	7.06	D_1_ (8.46); H_1_ (9.07)
Thioridazine	9.4	8.82	6.84	7.56	α_1A_ (8.5); α_1B_ (8.62); M_1_ (7.89); M_5_ (7.9); H_1_ (7.78)
Pimozide	9.48	9.6	6.19	7.32	5-HT_7_ (9.3); D_4_ (8.74)
Perphenazine	8.47	9.89	6.38	8.25	5-HT_6_ (7.77); 5-HT_7_ (7.64); D_1_ (7.52); D_4_ (7.77); α_1A_ (8); H1 (8.1); σ_1_ (7.73)
Loxapine	7.96	7.71	5.61	8.18	5-HT_2C_ (7.88); 5-HT_6_ (7.51); 5-HT_7_ (7.06); D_1_ (7.27); D_4_ (8.08); D_5_ (7.12); α_1A_ (7.51); α_1B_ (7.28); α_2A_ (6.82); α_2B_ (6.97); α_2C_ (7.1); M_1_ (6.92); H_1_ (8.3)
**ATYPICAL ANTIPSYCHOTICS**					
Clozapine	6.87	6.66	7.06	8.39	5-HT_1B_ (6.28)b; 5-HT_2B_ (8.79); 5-HT_2C_ (8.56 - IA); 5-HT_3_ (6.62); 5-HT_6_ (7.87); 5-HT_7_ (7.75); D_1_ (7.64); D_4_ (7.33); D5 (6.63); α_1A_ (8.79); α_1B_ (8.15); α_2A_ (7.43); α_2B_ (7.58); α_2C_ (8.22); M_1_ (8.21); M_2_ (7.44); M_3_ (7.72); M_4_ (7.81); M_5_ (7.81); H_1_(8.95); H_2_ (6.82); H_4_ (6.18)
Risperidone	8.21	8.16	6.75	9.69	5-HT_1B_ (7.83); 5-HT_1D_ (7.07); 5-HT_2B_ (7.8); 5-HT_2C_ (8.17); 5-HT_7_ (8.18); D_4_ (8.21); D_5_ (7.8); α_1A_ (8.3); α_1B_ (8.04); α_2A_ (7.78); α_2C_ (8.89); H_1_ (7.7)
Olanzapine	7.67	7.46	5.82	8.88	5-HT_2B_ (8.41); 5-HT_2C_ (8.41); 5-HT_3_ (6.69); 5-HT_6_ (8.09); 5-HT_7_ (6.98); D_4_ (7.75); D_5_ (7.04); α_1A_ (6.95); α_1B_ (6.58); α_2A_ (6.5); α_2B_ (7.09); α_2C_ (7.54); M_1_ (7.58); M_2_ (7.2); M_3_ (7.28); M_4_ (7.61); M_5_ (8.12); H_1_ (8.66); H_2_ (7.36)
Ziprasidone	8.09	8.35	9.01	9.51	5-HT_1B_ (8.4); 5-HT_1D_ (8.64); 5-HT_2B_ (9.08); 5-HT_2C_ (9.01); 5-HT_6_ (7.21); 5-HT_7_ (8.22); D_1_ (8.45); D_4_ (7.33); α_1A_ (7.74); H_1_ (7.2); SERT (7.26); NET (7.32)
Quetiapine	6.38	6.41	6.78	6.81	5-HT_2B_ (7.33); 5-HT_2C_ (5.98); 5-HT_6_ (6.02); 5-HT_7_ (6.51); D_1_ (6.71); D_4_ (5.85); D_5_ (7.8); α_1A_ (7.66); α_1B_ (7.84); α_2A_ (5.44); α_2C_ (7.65); H_1_ (8.16); H_2_ (7.38); M_1_ (489); M_3_ (5.79)
Brexpiprazole	9.48	8.9	9.21	9.67	α_1A_ (8.42); α_1B_ (9.23); α_1*D*_ (8.58); α_2C_ (9.77); 5-HT_2B_ (8.72); 5-HT_7_ (8.43)
Aripiprazole	8.9	8.85	8.57	8.02	5-HT_2B_ (9.59)
Cariprazine	9.31	10.07	8.59	7.73	5-HT_2B_ (9.24)
Amisulpiride	8.89	8.62	5.8	5.08	5-HT_2B_ (7.89); 5-HT7 (7.94)
Blonaserin	9.84	9.3	6.09	9.09	

The pK_i_ values shown are taken from several sources, including mean values from the PDSP K_i_ database (Roth and Driscol, 2016). The values for the receptors: D_2_, D_3_, 5-HT_1A_, 5-HT_2A_, 5-HT_2B_, 5-HT_2C_ with atypical antipsychotics were from Leggio et al. (2016) or Citrome (2015). The colors indicate: Blue, drugs with higher affinity for D_2_ receptors; Orange, drugs with a 5-HT/DA binding profile; Violet, drugs with a D2/3 effects; Green, drugs with partial agonist activities at D_2_ receptors. 5-HT_2B_ receptors are a recurrent target of several atypical antipsychotic drugs. Conversely, none of the agents presented above bind at 5-HT_4_ receptors. M1-M5, muscarinic receptors; σ1, sigma 1 receptors.

* in the range of 1 order of magnitude compared to the affinity at D_2_ receptors.

The superiority of clozapine in terms of low incidence of EPS has been also related to its low affinity at D_2_ receptors, implying a fast dissociation from the receptor and avoiding unsurmountable D_2_ receptor blockade, either acutely or chronically (Kapur and Seeman, 2001). This property has not been verified for all antipsychotics but it introduces the notion that antipsychotic drugs could correct rather than block central DA transmission. Aripiprazole brexpiprazole or cariprazine display partial agonist activities at some signaling pathways connected to D_2_ receptors (Kiss et al., 2010; Citrome, 2013, 2015). In spite of the high occupancy of D_2_ receptors *in vivo* presumably overwhelming the 80% threshold occupancy, these partial or biased agonists do not induce catalepsy in rats and induce fewer EPS in humans compared to typical neuroleptics (Citrome, 2015).

#### Questions and directions

There are several outstanding questions for neuropharmacology. In spite of the availability of antipsychotic drugs minimizing the occurrence of EPS, abnormal motor responses are still present with some of these medications.

**Figure 3:**
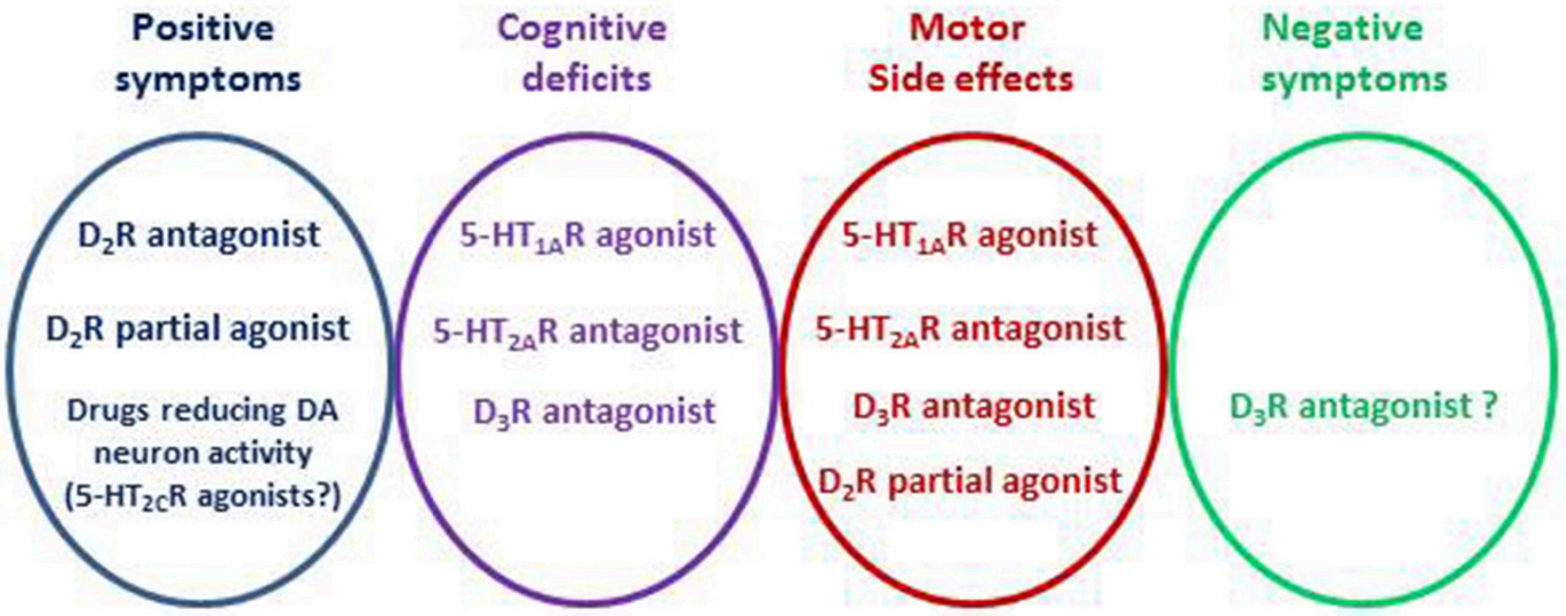
**Design of antipsychotic drugs**. The elaboration of antipsychotic drugs pays attention to the positive symptoms, negative symptoms, cognitive deficits and extrapyramidal side effects. The D_2_ receptor subtype is the main target for the positive symptoms. The 5-HT_2C_ receptor is an example of preclinical research target offering another possibility based on the reduction of DA neuron activity. Different targets are proposed to limit the other deficits or to avoid motor side-effects including the 5-HT_2A_, 5-HT_1A_ or D_3_ receptor subtypes. Nowadays, one of the main difficulties is to address the negative symptoms and some preclinical studies suggest beneficial effects of targeting the D_3_ receptor subtypes.

- The reason why 5-HT_2A_ receptor antagonism has beneficial effects is still not fully understood with respect to the lower EPS and the involvement of motor part of the basal ganglia. There are reasonable amounts of data showing that the impact of D_2_ receptor blockade in the presence of 5-HT_2A_ receptor antagonism would be reinforced in the mesolimbic system and conversely dampened in the mesocortical system (Meltzer and Huang, 2008). Indeed, 5-HT_2A_ receptor antagonists enhance DA release induced by D_2_ receptor blockade in the cortex. A similar property has been observed with 5-HT_1A_ receptor agonism (Meltzer and Massey, 2011; Hensler et al., 2013; Schreiber and Newman-Tancredi, 2014). This may explain the fact that antipsychotic drugs like cariprazine, with low affinity for 5-HT_2A_ receptor and moderate to high affinity for 5-HT_1A_ receptors, display atypical antipsychotic drug profiles in the clinic (Kiss et al., 2010; De Deurwaerdère, 2016). The favorable profile regarding motor parts of the basal ganglia is not likely to be related to a putative disinhibitory action of 5-HT_2_ receptor blockade on DA neuron activity, as had been previously speculated (Kapur and Remington, 1996; De Deurwaerdère and Di Giovanni, 2016). Rather, their cortical effects could have beneficial effects on cognitive symptoms of the disease that are, in part, independent from D_2_ receptor blockade. The categorization in typical vs. atypical antipsychotics is not always clear in considering only the binding profiles (Table 1).
- The negative symptoms remain one of the most difficult challenges in the treatment of schizophrenia (Millan et al., 2014; Remington et al., 2016). Interestingly, neuropsychopharmacological analysis tends to dissociate various symptoms of schizophrenia which could have distinct etiology (Buckley et al., 2009). Acting on these distinct dimensions implies a combination of selective drugs or the use of multi-target medicines (Butini et al., 2016), and animal models adapted for the different symptoms (Millan et al., 2014). This implies, in turn, an extensive preclinical evaluation for testing the suitability of any new compound (Millan et al., 2015b; De Deurwaerdère, 2016; Di Giovanni and De Deurwaerdère, 2016). Some of the most advanced treatments focus on the following targets (Table 1): D_2_ receptor antagonism regarding the positive symptoms, 5-HT_1A_ receptor agonism/5-HT_2A_ receptor antagonism and/or increase in cortical acetylcholine release for the cognitive symptoms, and perhaps D_3_ receptor antagonism for the negative symptoms (Figure 3).
- The pharmacological analysis of the newer antipsychotic drugs has led to consideration of other targets including 5-HT_6_, 5-HT_7_ receptors (Meltzer and Huang, 2008; Mauri et al., 2014) or the intriguing 5-HT_2B_ receptors (Devroye et al., 2016) (Table 1). Since the interaction between neurotransmitter systems is extremely complex, actions directed to some targets (e.g., D_2_ receptors) might unmask a deleterious involvement of other circuits. For instance, while the 5-HT_2C_ receptor blockade has not been shown to be primarily involved in the antipsychotic drug action, its blockade might be necessary to limit the occurrence of EPS induced by typical neuroleptics (Richtand et al., 2008). The neurobiological process underlying a deleterious involvement of 5-HT_2C_ receptors that follows D_2_ receptor blockade is not well understood (De Deurwaerdère et al., 2013). Because of the frequency of co-morbid diseases including mania, anxiety or depression in schizophrenic patients, this analysis further indicated the prescription of other medicines in addition to antipsychotic drugs (Buckley et al., 2009). Most available antipsychotic drugs have a complex pharmacological profile including adrenergic, muscarinic and histaminergic sites and most of them, especially the newer ones, are also developed for the treatment of bipolar disorder and major depression.
- Very few strategies have tried to avoid the D_2_ receptor target because most, if not all trials targeting a single site other than D_2_ receptors failed to reach clinical significance (Deutch et al., 1991; Ashby and Wang, 1996; Meltzer and Massey, 2011). Recently, the 5-HT_2C_ receptor agonist vabicanserin has been tested as a monotherapy in the treatment of schizophrenia. This was justified as the 5-HT_2C_ receptor inhibits DA release, at least in the nucleus accumbens, and its preferential agonists are efficacious in several preclinical models of schizophrenia (Rosenzweig-Lipson et al., 2012). Although the strategy of stimulating 5-HT_2C_ receptors should be taken with caution (Di Giovanni and De Deurwaerdère, 2016), it protects at least from the development of obesity due to its anorexigenic property and would be less prone to promoting EPS. Although having an antipsychotic profile, vabicanserin was not more efficacious than risperidone at primary end point, and its development was discontinued (Shen et al., 2014). Nonetheless, vabicanserin might represent a change of strategy regarding the development of newer antipsychotics, focusing on the activity of the monoaminergic systems rather than on a single site.

#### Conclusion

After having isolated one target of antipsychotic drugs, namely the D_2_ receptor subtype, the search for additional targets is essential for better intervention of the full spectrum of symptoms in schizophrenia. This cannot be achieved by acting on a single target, necessitating the use of a cocktail of drugs or multi-targeted compounds (Figure 3). The methodological approach to developing antipsychotic drugs is still conditioned by molecular pharmacology, although new developments in connectomics should prove valuable in the future (Fornito and Bullmore, 2015).

### Treatments of depression

#### Historical perspectives of antidepressant drugs

Major depression is a severe, highly disabling, psychiatric disorder which is becoming a leading cause of global disease burden, with a growing prevalence of 3–17% in adults and 2.8–5.6% in adolescents (Gururajan et al., 2016; Rantamaki and Yalcin, 2016). Its multifaceted, but poorly understood etiology involves complex interactions between different biological (genetic, epigenetic, neuroendocrine and neuroimmune), psychosocial (personality traits), developmental and environmental (exposure to early life stress) factors.

In the 1950s began a new era in the field of psychopharmacology with the clinical introduction of two antidepressants: iproniazid and imipramine (López-Muñoz and Alamo, 2009). Iproniazid, originally developed for anti-tuberculous purposes, was characterized as MAO inhibitor (MAO-I) by Zeller and Barsky (1952). Its administration improved mood in patients and it was shown to lead to an increase in the brain levels of 5-HT in rodents (Udenfriend et al., 1957). The discovery of the antidepressant effects of iproniazid laid the path for development of more effective MAOIs such as phenelzine, tranylcypromine, isocarboxazid, and other hydrazine and indole derivates (Ban, 2001). The compound, now known as imipramine, was presented by Kuhn in 1957 as the first representative of the tricyclic antidepressants (TCAs) (Brown and Rosdolsky, 2015). In the period between 1960 and 1979, many different TCAs were developed, including amitriptyline, nortriptyline, desipramine, trimipramine, protryptyline, iprindol, dothiepin and clomipramine (Fangmann et al., 2008; López-Muñoz and Alamo, 2009). First tricyclic antidepressants were shown to inhibit the uptake of NA. The modification of the basic dibenzazepine structure of imipramine resulted in the discovery of new cyclic antidepressants during 1970s, including maprotiline and mianserine. The significant therapeutic effects of MAO-Is and TCAs, stimulated an increased interest in the role of monoaminergic system in the etiology of depression.

Fluoxetine, the first selective serotonin reuptake inhibitor (SSRI), came out in 1975 (Wong et al., 1975) and was introduced in 1987. Its unique properties and appropriate clinical efficacy established the SERT as an important target in the treatment of depression (Perez-Caballero et al., 2014). Fluoxetine is, in fact, not highly selective toward the SERT whereas citalopram, escitalopram, fluvoxamine, paroxetine or sertraline block the SERT more selectively (Thomas et al., 1987; Jacquot et al., 2007). Their efficacy is similar to TCAs but SSRIs generally have lower toxicity in comparison with MAO-Is and TCAs.

#### Mechanisms of action of antidepressant drugs

The neurobiological basis of depression is not fully understood although the monoaminergic theory of depression stipulates 5-HT and NA extracellular deficits in different brain regions. This remains far from to be clear however, except in major depression with suicidal behavior where the levels of tissue 5-HT were found lower compared to levels in age-matched controls (Delgado and Moreno, 2000). In animals, the destruction of either 5-HT or NA systems, or both, does not trigger depressive-like symptoms in some behavioral tests (Page et al., 1999; Cryan et al., 2002, 2005; Delaville et al., 2012). On the other hand, the enhancement of 5-HT and NA neurotransmission with some antidepressant medication results in the improvement of the common symptoms of the disease.

Studies with SSRI and other blockers of monoaminergic reuptake sites indicate that the efficacy of antidepressant drugs entails at least three sequential steps. (1) The blockade of SERT at the level of 5-HT cell bodies enhances somatodendritic 5-HT extracellular levels (Bel and Artigas, 1992; Invernizzi et al., 1992), thereby activating 5-HT_1A_ autoreceptors and reducing the firing rate of 5-HT neurons (Blier and de Montigny, 1990; Adell et al., 1993; Guiard et al., 2005). The blockade of SERT at terminals of 5-HT neurons enhances extracellular levels of 5-HT but this effect is considerably dampened by the decrease in 5-HT neuron firing rate and the stimulation of 5-HT_1B_ autoreceptors located at 5-HT terminals (Invernizzi et al., 1992; Gardier et al., 2003). (2) The desensitization of somatodendritic 5-HT_1A_ receptors in the raphe nuclei upon subchronic administration of SSRI, with the main consequence of magnifying the effects of the SERT blockade on the extracellular levels of 5-HT (Invernizzi et al., 1996; Piñeyro and Blier, 1999). (3) The final step involves modification of 5-HT receptors at 5-HT receptive cells including 5-HT_1A_, 5-HT_2A_ or 5-HT_2C_ receptors (Artigas et al., 2002; Artigas, 2013). These sequential steps take time which may account for the delay between start of treatment with antidepressant drug and clinical effect.

The interaction between 5-HT and NA systems is evident in the mechanism of action of TCAs because most TCAs have a better affinity for the NET compared to the SERT. Numerous studies have provided evidence that α_1_ and α_2_ receptors exerted complex interactions on 5-HT neurons, α_1_ receptor being excitatory. Conversely, 5-HT at least via 5-HT_2A_ receptors inhibits the activity of NA neurons whereas the desensitization of 5-HT_2A_ receptors, also occurring after SSRI, allows for an increase in central NA tone (Piñeyro and Blier, 1999; Hamon and Blier, 2013). Thus, the interaction between the 5-HT and the NA has a pivotal role in the mechanism of action of drugs targeting monoaminergic neurons terminal activity including TCAs, SSRI and MAO-I. Other antidepressant drugs target 5-HT and/or NA receptors (see below), but their mechanisms of action are less well understood. Through the interaction between monoaminergic systems (Hamon and Blier, 2013), DA changes are indirectly involved in the mechanism of action of antidepressants. The DA system is usually not directly targeted in the treatment of depression (nonetheless, see below with the introduction of antipsychotics in major depression).

#### Amelioration of antidepressant drugs actions

In the last 20 years two main problems with antidepressant treatment have emerged, including a poor or partial response, i.e., a large proportion of patients not responding well to the therapy (Gumnick and Nemeroff, 2000), and the delay of action of antidepressant drugs usually reached after 2–3 weeks of treatment. The partial response of antidepressant drugs has led to the introduction of new and more effective antidepressants like venlafaxine, which inhibits the reuptake of 5-HT and NA (NSRI), and mirtazapine, which acts primarily as an antagonist of presynaptic α_2_-adrenoreceptors (Artigas et al., 2002). A meta-analysis assessed the effects of 12 new-generation antidepressants and showed that mirtazapine, venlafaxine and two SSRIs, escitalopram and sertraline, were more efficacious than other SSRIs (fluoxetine, fluvoxamine and paroxetine), the NSRI duloxetine and the selective NA reuptake inhibitor (SNRI) reboxetine (Cipriani et al., 2009).

The antidepressant effect of MAO-Is is dependent on inhibition of MAO-A but irreversible inhibition of that isoenzyme resulted in adverse hypertensive reactions with foods containing tyramine called “the cheese effect.” To avoid this adverse event, a new strategy for the treatment of depression was presented in the form of reversible inhibitors of MAO-A (RIMA), such as moclobemide (Haefely et al., 1992), brofaromine (Davidson, 2003), and befloxatone (Curet et al., 1998). However, the use of MAO-Is today is mostly limited for treating patients with atypical depression, bipolar depression, treatment-resistant depression, or hyperserotonergic responses with SSRIs. In 2009, agomelatine, the first antidepressant that mediates its activity via the melatonergic pathway was approved (San and Arranz, 2008; Millan et al., 2010; Racagni et al., 2011). This antidepressant acts as an antagonist at 5-HT_2C_ receptors and agonist at melatonergic MT_1_ and MT_2_ receptors (Millan et al., 2010). In comparison with other standard SSRIs and SNRIs, agomelatine showed similar or even favorable efficacy and tolerability (Kasper et al., 2013). In addition to agomelatine, several other 5-HT_2C_ agents have been reported to display strong anxiolytic/antidepressant properties in animal models and clinical studies (Millan, 2005, 2006; Millan et al., 2005; Di Giovanni and De Deurwaerdère, 2016). A summary of these evolutions has been indicated in the Table 2.

**Table 2:** **Pharmacological targets of lead and newer antidepressant drugs from lead compounds**.

**Lead compounds**	**Mechanisms of action**	**New compounds in clinic**	**Main targets**
Imipramine, Fluoxetine	5-HT/NA interaction mostly via blockade of SERT and/or NET + Alteration of monoamine targets and function	SSRI (Escitalopram, Citalopram, Paroxetine…) NSRI (Duoxetine, Venlafaxine) SNRI (Reboxetine) Multi-target drugs (Vortioxetine)	SERT SERT and NET NET SERT/5-HT receptors
Iproniazid	5-HT/NA interaction mostly via inhibition of	Moclobemide	MAO-A (reversible inhibitor)
	MAO-A/B (irreversible)	Agomelatine	MT1/MT2 agonist/5-HT_2C_ antagonist

Lead antidepressant drugs and their proposed mechanisms of action as well as newer drugs in clinical use that retain some of their targets. Agomelatine is included, although its 5-HT_2C_ receptor antagonist action was not directly based on a lead drug (Di Giovanni and De Deurwaerdère, 2016).

The second problem associated with antidepressant treatment is the delay of action of the drugs. Knowledge of the mechanism of action of SSRIs resulted in the proposal to reduce the delay of action by blocking 5-HT_1A/1B_ autoreceptors, thereby lessening the time period needed to produce the massive increase in 5-HT levels at the synaptic cleft (Artigas et al., 1996). However, since the efficacy of SSRI is also associated with postsynaptic 5-HT_1A_ receptors (Blier et al., 1990), which are not desensitized and are required for the benefit of SSRIs, such a blockade might be expected to be counterproductive. Similar augmentation can be achieved by concomitantly blocking 5-HT_2C_ receptors, which indirectly inhibit 5-HT neuronal activity (Cremers et al., 2004, 2007). Clinical trials involving this approach are underway (see Di Giovanni and De Deurwaerdère, 2016). Another monoaminergic possibility to obtain a fast onset of the antidepressant effect is the use of 5-HT_4_ receptor agonists. These compounds act directly on 5-HT receptive fields in the hippocampus or the cortex and indirectly stimulate 5-HT release. Their pro-cognitive and anxiolytic effects have raised some interest for the treatment of depression (Lucas and Debonnel, 2002; Lucas et al., 2007; Mendez-David et al., 2014).

#### Future directions

Newer antidepressants have been developed with more complex pharmacological profile such as vortioxetine (Sanchez et al., 2015). This compound blocks the SERT as well multiple 5-HT receptors (Sanchez et al., 2015; Sagud et al., 2016). This effect is in line with studies reporting that the combination of antidepressants with atypical antipsychotic drugs (olanzapine, risperidone, quetiapine, aripiprazole, paliperidone, ziprazidone, and amisulpride), can boost the effectiveness of depression treatment (Berman et al., 2007; Thase et al., 2007; Bauer et al., 2009; Han et al., 2013). Thus, the monoaminergic system remains an important field for producing newer antidepressant drugs involving refined strategies (Hamon and Blier, 2013; Artigas, 2015). Recently, there has been a growing interest in neuronal plasticity, cholinergic, GABAergic and glutamate neurotransmission, stress and HPA axis, reward system and neuroinflammation as potential underlying mechanisms of depression that go somehow beyond monoaminergic strategies (Artigas, 2015; Dale et al., 2015; Millan et al., 2015a). However, the complexity of the system is such that monoaminergic antidepressant drugs may act on different neurobiological circuits and molecular mechanisms, which might be more important targets.

### Obesity and energy balance

#### Historical perspective

The neurobiological networks involved in food intake and energy balance have been the subjects of many recent studies, in view of the growing evidence for a worldwide problem of obesity. The first anti-obesity drug targeting amine neurotransmitter signaling was amphetamine. Amphetamine, α-methylphenethylamine, member of β-phenylethylamine group of drugs, was synthesized to substitute for ephedrine. Animal and human studies showed that this compound can produce arousal and insomnia. Racemic α-methylphenethylamine, registered by SK&F under the name Benzedrine®, was introduced in 1935 to treat narcolepsy and was also employed for mild depression, post-encephalitic parkinsonism, schizophrenia, alcoholism and other disorders (see (Bett, 1946)). In 1937, SK&F marketed Dexedrine (d-amphetamine), the more potent of the two isomers which produced euphoria, excitability, hyperactivity and restlessness with hyperthermia. The quick loss of weight in obese subjects treated against narcolepsy with Benzedrine aroused the interest in the use of the sympathomimetics as anti-obese medicines. A clinical study on 162 patients treated with d-amphetamine, which had been performed on a smaller number of subjects, supported its usefulness as appetite depressant (Hawirko and Sprague, 1946). However, d-amphetamine produces addiction (Kiloh and Brandon, 1962). To decrease addictive potential, substituted amphetamines such as phenyl-tertiary-butylamine, phentermine, and fenfluramine (3-trifluoromethyl-N-ethylamphetamine) were synthesized and were approved by FDA between 1959 and 1973 for weight loss.

#### Mechanism of action

The hypothalamus is a key structure involved in food intake regulation and energy balance. It receives monoaminergic terminals from several different sources and the roles of the monoamines appear to be different: 5-HT and histamine participate as a satiety factors; DA acts as the main signaling molecule in a reward system, and NA tends to enhance energy expenditure. Animal studies have shown that phenyl-tertiary-butylamine, phentermine, and fenfluramine cause the release of amines from the nerve terminals as well as inhibiting their uptake. While d-amphetamine has higher affinities for DAT and NET than SERT, fenfluramine has a higher affinity for SERT (Garattini, 1995; Kuczenski et al., 1995). In rat, both peripheral and central mechanisms of fenfluramine action have been implicated (Carruba et al., 1986).

Studies with selective ligands of 5-HT receptors *in vivo* have shown that hypophagic effect triggered by d-fenfluramine and its metabolite d-norfenfluramine was mediated by 5-HT acting on 5-HT_2C_ receptors (Vickers et al., 2001) (Table 3). Combination of phentermine and fenfluramine, i.e., “Phen-Fen,” enables the therapeutically effective dose of appetite suppressants to be decreased. However, the heart valve disease related to a 5-HT_2B_ receptor agonism action and pulmonary hypertension (Vivero et al., 1998; Palmieri et al., 2002) caused the withdrawal of fenfluramine and dexfenfluramine from the U.S. market in 1997. Later meta-analysis of the observational studies indicated that fenfluramine-associated valvular regurgitation was less common that assumed, but still present in 1 out of 8 patients treated for >90 days (Sachdev et al., 2002). The next anti-obesity drug sibutramine (Luque and Rey, 1999), originally developed as an antidepressant, is a potent SERT and NET inhibitor *in vivo* (Luscombe et al., 1990). Animal studies on its mechanisms of action pointed to the activation of β_1_-adrenoceptors, 5-HT_2A/2C_ receptors and α_1_-adrenoceptors (Jackson et al., 1997). The compound has been shown in clinical trial to be an efficient therapeutic, although the patients who stopped using the drug quickly regained weight (James et al., 2000), as observed for the substituted amphetamines (Vivero et al., 1998). Sibutramine, having antiatherogenic activities (improvement of insulin resistance, glucose metabolism, dyslipidemia, and inflammation) concomitantly induces a moderate increase in heart rate, a slight increase in blood pressure and may also prolong the QT interval, inducing arrhythmias (Scheen, 2010). Because the hazard ratio for cardiovascular events was significantly increased in sibutramine treated patients, the drug was withdrawn from markets in 2010 (Haslam, 2016).

**Table 3:** **Anti-obesity drugs and mechanisms**.

**Lead compounds**	**Mechanisms of action**	**New compounds in clinic**	**Main targets**
Amphetamine	DAT, NET and release of catecholamines	Sibutramine Reboxetine	NET/SERT (withdrawn) NET
d-fenfluramine	5-HT release and indirect 5-HT_2C_ agonism	Lorcaserin	Preferential 5-HT_2C_ receptor agonist
Antipsychotic drugs	Obesity as side effects via H_1_ receptor antagonism	Cariprazine, Brexpiprazole, Aripiprazole	Weak to no affinity toward H_1_ (even 5-HT_2C_) receptors (Table 1)

Lead compounds in the field of anti-obesity drugs and their proposed mechanisms of action are shown, together with newer drugs with similar actions. Amphetamine enhances catecholamines release through its action on DAT and NET, which led to the development of drugs that enhanced NA release. D-fenfluramine, chemically derived from amphetamine, loses affinities for DAT and NET in relation to SERT. Conversely, antipsychotic drugs induced weigh gain that has been corrected in newer compounds.

In 2012, a novel drug, lorcaserin, was approved by FDA for the treatment of obesity. It is a 5-HT_2C_ receptor agonist that is appetite suppressing while free of cardiovascular effects (Gustafson et al., 2013). It has about 15 and 100 times higher affinity for 5-HT_2C_ than for 5-HT_2A_ and 5-HT_2B_ receptor subtypes. This drug stimulates the 5-HT_2C_ receptors located on proopiomelanocortin neurons in the arcuate nucleus resulting in the release of α-melanocortin-stimulating hormone, which suppresses appetite by acting on melanocortin-4-receptors in the paraventricular nucleus (Voigt and Fink, 2015). It probably has other actions in the brain including the decrease in DA release in the nucleus accumbens, dampening goal-directed behavior (Higgins and Fletcher, 2015; Di Giovanni and De Deurwaerdère, 2016).

#### Histamine and obesity

The H_1_ receptor antagonist component of various medications including antipsychotics is suspected to increase body weight (Kroeze et al., 2003; Ratliff et al., 2010). In addition, recent work indicates also that cerebral histaminergic system mediates the anorexic effects of oleoylethanol amide, an endogenous agonist at peroxisome proliferator-activated receptor alpha (PPAR-α) (Provensi et al., 2014). Experimental and preclinical studies employing different animal models have convincingly shown that H_1_ and H_3_ receptors play an important role in energy balance and body weight gain, H_1_ receptor agonists and H_3_ receptor antagonist/inverse agonists being anorexic drugs (Clineschmidt and Lotti, 1973; Lecklin et al., 1998; Masaki et al., 2004; Provensi et al., 2014, 2016).

Although much attempt has been done in developing histaminergic agents as anti-obesity treatment, no recent drug with a histaminergic profile reached clinics (Esbenshade et al., 2006; Provensi et al., 2016). Betahistine, registered in Europe in 1970 for the management of vertigo and vestibular pathologies, could be used to achieve anti-obesity action in some conditions (Provensi et al., 2016). This drug, combining weak agonism at H_1_ receptor and inverse agonism at H_3_ receptor, stimulates histamine synthesis and release in tuberomammillary nuclei of the posterior hypothalamus (Arrang et al., 1985; Tighilet et al., 2002; Gbahou et al., 2010). On the one hand, only 10% of 281 obese participating to a multicenter randomized placebo controlled trial positively responded to the 12 weeks therapy with betahistine (Barak et al., 2008). On the other hand, betahistine was shown to limit the excess body weight induced by the antipsychotic drug olanzapine (Table 1) in a randomized, double blind placebo controlled pilot study involving 36 patients with either schizophrenia or schizoaffective disorders (Barak et al., 2016). In an earlier double blind placebo-controlled study that involved similar number of enrolled patients, betahistine potentiated the anorexic effect of the NET blocker reboxetine in patients receiving olanzapine (Poyurovsky et al., 2013).

#### Conclusions regarding monoamines and obesity

The study of the mechanism of action of d-fenfluramine led to completely new drugs which is an interesting achievement. Additional data are warranted in the field of the control of energy balance and monoaminergic targets. The reduction of BMI in obese patients with lorcaserin is estimated in the range of 5–10%. This is less compared to the actions of previous drugs amphetamine, d-fenfluramine or sibutramine, but probably safer. Regarding antipsychotic drugs, newer antipsychotics (aripiprazole, cariprazine) display a lower affinity at H_1_ receptor to prevent weight gain as recommended earlier (Kroeze et al., 2003). A summary has been indicated in Table 3.

## From pathophysiology to treatments

### Parkinson's disease

#### Historical perspectives

PD is a neurological condition characterized by motor impairments including bradykinesia, tremor and rigidity (Olanow et al., 2009). The symptoms are a consequence of the degeneration of the nigrostriatal DA tract and appear approximately when 30% of DA cells in the SNc are destroyed and/or when striatal tissue DA is reduced by 70% (Burke and O'Malley, 2013). Comorbid diseases are noticed with prevalence ranging from 30 to 45% in the case of anxiety or depression (McDonald et al., 2003). The etiology of the comorbid diseases is still unknown although other neuronal changes have been identified including alterations in 5-HT and NA neurons (Jellinger, 1991; Galati and Di Giovanni, 2010; Delaville et al., 2011). More generally, PD is a synucleopathy characterized histopathologically by the presence of Lewy bodies in multiple brain regions including the SNc and the LC. The lesions of LC NA neurons occur earlier than SNc DA neurons, and can reach a higher extent (Del Tredici et al., 2002; Rommelfanger and Weinshenker, 2007; Delaville et al., 2011). The loss of NA input to the nigrostriatal DA system could be detrimental for the function of DA neurons (Benarroch, 2009), conferring to NA system both a protective role on DA neurons and, consequently a positive influence regarding the outcome of treatments. However, available treatments mostly target DA and not NA-related mechanisms (Table 4).

**Table 4:** **Antiparkinsonian drug treatments and pharmacological target**.

**Pathophysiology**	**Drug in the market**	**Mechanism of action**	**Efficacy at pharmacological target**
Degeneration of SNc DA neurons and striatal DA loss	L-DOPA DA receptor agonists	In part related to DA increase in the brain, possibly outside the striatum Stimulation of D_2/3_ receptors	Unresolved Unresolved
Degeneration of LC NA neurons	None		
Degeneration of neurons—oxidative stress	MAO-B inhibitors (Rasagiline)	Unclear	Unresolved

The table represents brain alterations in Parkinson's disease and the drugs and/or strategies used against these alterations. The full spectrum of the mechanism of action of these drugs may have been misunderstood as no clear efficacy at pharmacological target has been achieved.

The treatments of PD depend on the stage of the disease. L-DOPA was the first treatment aimed at restoring DA transmission in the brain. This amino acid can cross the blood brain barrier (BBB) and reach the striatum before conversion to DA by AADC. The first trials started in 1961 and L-DOPA still represents the main pharmacological treatment of PD (Lees et al., 2015). Years later it was followed by the administration of DA receptor agonists, and both treatments correspond to the so-called DA replacement therapy. Deep brain stimulation of the subthalamic nucleus is a second therapeutic option in advanced stages of the disease, where patients respond poorly to medication with motor complications and have no psychiatric antecedent (Bronstein et al., 2011). A commonly used option at the onset of the disease, which appears to have few unwanted side-effects, consists in administration of MAO-B inhibitors such as L-deprenyl or rasagiline (Rascol et al., 2011; Hauser et al., 2016).

While the side-effects are still debated in the case of deep brain stimulation of the subthalamic nucleus (Bronstein et al., 2011), long-term pharmacological treatments are associated with severe motor and psychiatric side effects (Bastide et al., 2015).

#### Mechanisms of action of antiparkinsonian drugs

The therapeutic benefit of L-DOPA and DA receptor agonists has been related to their indirect and direct actions, respectively, at DA receptors. L-DOPA has been shown to enhance extracellular levels of DA in animal models of the disease, using *in vivo* methods or in humans indirectly measured by the displacement of PET ligands binding at DA receptors (de la Fuente-Fernández, 2013; De Deurwaerdère et al., 2016). Since the enzyme AADC is present in most tissues as well as in brain, much of the administered L-DOPA was converted at the periphery to DA, which does not cross the BBB. This problem was ameliorated by the introduction of benserazide or carbidopa, two peripheral AADC inhibitors that do not penetrate the brain (Cotzias, 1968; Cotzias et al., 1969). The introduction of COMT inhibitors may also increase the efficacy of L-DOPA by limiting its transformation into 3-O-methyl-DOPA, and, perhaps, by limiting the inactivation of central DA (Ries et al., 2010). One of the main side effects of L-DOPA therapy, namely L-DOPA-induced dyskinesia, has been related to the variation of plasma L-DOPA concentrations inherent to its oral administration. The pharmacokinetic challenge is to find a means of smoothing its plasma concentrations. Similarly, a continuous stimulation of DA receptors with agonists is an important criterion, and selective agonists with long-lasting action have been recently synthesized (Butini et al., 2016).

No real progress has been made regarding the pharmacodynamics of L-DOPA: researchers attempted either to boost the ability of L-DOPA to release DA in the striatum during the 1980's and the 90's, or, more recently, to limit the excess of striatal DA release thereby reducing the occurrence of dyskinesia. The danger behind these strategies, which focused on a putative restoration of striatal DA release, magnified by the implantation of grafts aimed at releasing DA into the caudate-putamen of patients, is that nobody knows the extent to which striatal DA release is involved in the benefit and side-effects of peripheral L-DOPA (De Deurwaerdère et al., 2016). We now know that L-DOPA-induced dyskinesia and motor benefit can occur without a measurable enhancement of striatal DA release in animals (Porras et al., 2014), which confirmed earlier data raising doubt about the role of striatal DA in the mechanism of action of L-DOPA (Nakazato and Akiyama, 1989; Fisher et al., 2000).

In humans, the displacement of raclopride binding was observed after the intravenous administration of 3 mg/kg L-DOPA (de la Fuente-Fernández et al., 2004). The authors reported the displacement of ^11^C-raclopride binding, but indicated also that the presumed levels of extracellular DA produced by the i.v. administration of L-DOPA were lower in patients compared to healthy individuals (de la Fuente-Fernández, 2013). A similar situation occurs in rodent models at comparative, but behaviorally efficacious dosage (Navailles et al., 2010). The conclusion is that the role of striatal DA release may have been overestimated in the benefit and possibly in some side-effects elicited by L-DOPA (De Deurwaerdère et al., 2016).

**Figure 4:**
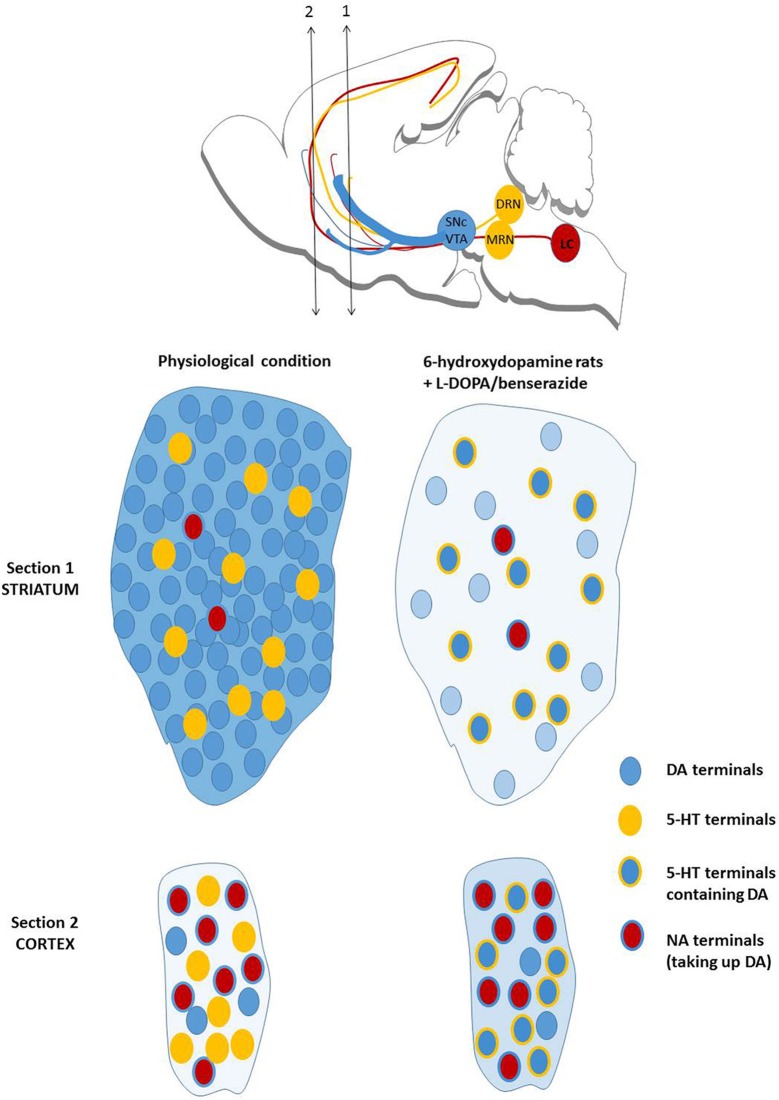
**Mechanisms of action of L-DOPA on brain DA release**. The upper illustration recalls the origin of ascending fibers for monoamines. The lower displays the normal dopaminergic transmission in the striatum (very dense) and the prefrontal cortex (very low). It includes serotonergic and noradrenergic terminals with their relative density compared to DA terminals. In the 6-hydroxydopamine rat model of PD, the density of dopaminergic fibers drop to less than 10% of the normal situation and the increase in DA release induced by L-DOPA is mostly due to serotonergic terminals. DA reuptake by noradrenergic fibers is low in the striatum due to their poor density. The overall output of striatal DA induced by L-DOPA, identified in the figure by the blue background, is very low compared to the physiological situation without L-DOPA. In the prefrontal cortex, the overall output of DA induced by L-DOPA is higher compared to the physiological situation because the density of serotonergic terminals is higher than the natural density of dopaminergic terminals. The reuptake of DA by NA fibers is magnified in L-DOPA-treated animals. The situation described in the prefrontal cortex is also observed in the hippocampus or the substantia nigra pars reticulata (not shown here) and virtually in most brain regions (De Deurwaerdère and Di Giovanni, 2016; De Deurwaerdère et al., 2016).

The overall picture is complicated by the fact that 5-HT neurons are mainly responsible for the release of DA induced by L-DOPA in animal models of the disease. The output of DA is dependent on the integrity of 5-HT neurons (Tanaka et al., 1999; Navailles et al., 2010). Due to the widespread innervation of 5-HT neurons in the brain, the first consequence is that L-DOPA-stimulated DA release occurs widely in the brain (Navailles et al., 2011b; Navailles and De Deurwaerdere, 2012a) (Figure 4), and this creates a new balance of central DA chemistry (Navailles and De Deurwaerdère, 2012b). The second consequence is that striatal DA released by L-DOPA is extremely low for at least three reasons: (i) the density of striatal 5-HT fibers is 10 to 20 times lower compared to the natural density of DA fibers; (ii) 5-HT neurons fire around 1 Hz or below and L-DOPA does not enhance the firing rate of 5-HT neurons (Miguelez et al., 2016b); DA neurons normally fire above 4 Hz (Bunney et al., 1973; Harden and Grace, 1995); (iii) the intraneuronal and neurochemical organization of striatal DA neurons (low metabolism, high rate of translocation into exocytotic vesicles) is specific to the DA terminals of the striatum and is not encountered in 5-HT terminals (De Deurwaerdère et al., 2016). The apparent higher magnitude of response to L-DOPA in the striatum compared to other brain regions simply corresponds to the stronger loss of clearance in the striatum due to the loss of DAT while the clearance of extracellular DA in extrastriatal regions is in part related to the NET (Navailles et al., 2014) (Figure 4).

The mechanism of action of L-DOPA is thus unclear and is likely different from DA agonists. Indeed, the efficacious agonists in the treatment of PD preferentially target the D_2/3_ receptors whereas L-DOPA favors D_1_ receptor-mediated mechanisms (Millan, 2010; Butini et al., 2016).

#### Amelioration of L-DOPA therapy

There remains scope for improvement in several aspects of L-DOPA therapy in terms of motor side effects, mood disorders, psychiatric side-effects and the evolution of the disease.

It has been proposed that excessive swings of extracellular DA are involved in the dyskinesia induced by L-DOPA (Carta et al., 2007), although this might not occur in the striatum as previously claimed. This possibility is supported by the finding that drugs or conditions that are able to decrease 5-HT neuronal activity reduced L-DOPA-induced dyskinesia (Bastide et al., 2015; De Deurwaerdère et al., 2016). Indeed, the mixed 5-HT_1A/1B_ receptor agonist, 5-HT_2C_ ligand, eltoprazine has been shown to reduce L-DOPA-induced dyskinesia in humans and animal models of the disease (Bezard et al., 2013; Svenningsson et al., 2015; Tronci et al., 2015). The efficacy of eltoprazine is not superior to existing treatment such as amantadine (Bezard and Carta, 2015). In terms of new strategies, particular attention has been given to 5-HT_1A_ receptor biased agonists, such as NLX-112, which acts preferentially at somatodendritic, rather than post-synaptic, 5-HT_1A_ receptors (Iderberg et al., 2015; McCreary et al., 2016). It has also been proposed that SSRIs lower L-DOPA-induced dyskinesia, as a result of their ability to indirectly reduce the firing rate of 5-HT neurons in the DRN (see above for the mechanism of action). The efficacy of SSRIs against L-DOPA-induced dyskinesia in humans is modest (Durif et al., 1995; Mazzucchi et al., 2015), and this is also found in animal models of PD (Fidalgo et al., 2015; Miguelez et al., 2016a). Indeed, higher doses of SSRIs are required, compared to those effective in normal individuals. This may be due to the action of L-DOPA inside the 5-HT neurons, impairing their normal biochemical processes and altering 5-HT release (Miguelez et al., 2016b), thereby dampening the reactivity of 5-HT neurons to SSRIs administration (Miguelez et al., 2016a). Other strategies that have been tested include D_3_ receptor antagonists or partial agonists (Bézard et al., 2003; Visanji et al., 2009). In addition to L-DOPA-induced dyskinesia, the D_3_ receptor subtype remains an important target in the treatment of PD with D_2/3_ receptor agonists (pramipexol, piribedil, ropinirole) (Millan, 2010; Leggio et al., 2016; Perez-Lloret and Rascol, 2016) being assessed in the management of impulsive-control disorder consequent to L-DOPA/DA receptor agonist administration (Seeman, 2015). Noradrenergic compounds have also been proposed to limit the occurrence of dyskinesia but the data are unclear (Delaville et al., 2011; Fox, 2013; Bhide et al., 2015; De Deurwaerdère et al., 2016).

The mechanism of action of L-DOPA toward 5-HT neurons also informs the therapeutic strategies for the mood disorders in PD, whose prevalence is higher compared to depressed individuals without PD (McDonald et al., 2003). Nowadays, the use of SSRIs remains a first line treatment in PD but, due to the specific action of L-DOPA on 5-HT neuron activity, the use of SSRIs might not be the best approach (Liu et al., 2013; De Deurwaerdère and Ding, 2016). Other strategies, based on the blockade of post-synaptic 5-HT or NA receptors, MAO-Is, SNRIs or DA receptor agonists, are available. An entire field of research has to be devoted to addressing this question, and also considering that the etiology of mood disorders in Parkinson's disease could be different from classical depression.

The psychotic side effects of DA agonists and L-DOPA have a high prevalence in advanced stage of the disease (Melamed et al., 1996). Historically, these effects have been associated with an excessive DA tone produced by L-DOPA or DA agonists. Interestingly, the 5-HT_2A_ receptor inverse agonist primavanserin has been shown to be effective in reducing L-DOPA-induced psychosis (Meltzer et al., 2010; Markham, 2016). It might replace the use of atypical antipsychotics quetiapine or clozapine (Friedman and Factor, 2000; Chang and Fox, 2016). The mechanisms behind the efficacy of primavanserin are, as yet, unclear.

Finally, MAO-B inhibitors L-deprenyl, safinamide, zonisamide or rasagiline have been shown to be efficacious in the treatment of PD either as unique treatment in the early stages of the disease or in conjunction with L-DOPA (Youdim et al., 2006; Riederer and Laux, 2011; Fox, 2013; Finberg, 2014). Since MAO-B is not primarily involved in the presynaptic inactivation of DA, it is possible that these compounds act at the glial level during L-DOPA therapy. In this context, L-DOPA has been shown to destroy 5-HT neurons upon chronic exposure in rodents (Navailles et al., 2011a; Stansley and Yamamoto, 2013, 2014), an effect related to an increase in metabolic stress which is blocked by antioxidant drugs including MAO-B inhibitors (Stansley and Yamamoto, 2014). The reduction of oxidative stress by MAO-B inhibitors might also reduce the destruction of DA neurons. MAO-B inhibitors of this type have also been shown to have antiapoptotic actions in a number of model systems (see (Youdim et al., 2006). Thus, the effects of these compounds in PD may be multifactorial.

#### Conclusion

The mechanisms behind the DA replacement therapy are unclear, because the mechanism of action of L-DOPA is still not understood in terms of its neurochemical effects, which differ from those of DA receptor agonists or MAO-B inhibitors (Table 4 for a summary). Therefore, the antiparkinsonian agents do not work as it was initially thought and this could explain why no major progress in terms of pharmacodynamics has been made. In the case of L-DOPA, it will be necessary to determine the roles of DA and the brain location of its effects, in order to regionally control DA tone from 5-HT neurons (Navailles and De Deurwaerdère, 2012b; Navailles et al., 2015).

Several models using transgene animals or injection of viral constructs targeting proteins involved in the familial forms of the disease have been raised during recent years (Bezard and Przedborski, 2011). Although these models are still under pathophysiological examination, they might open new ways of treatments, even beyond monoamines.

### Drug addiction

#### Historical perspective

The addictive properties of numerous drugs including alcohol and tobacco, the opiates (opium, morphine, heroin) and the psychostimulant drugs amphetamine and cocaine, were recognized before the existence and significance of the DA reward system in the brain was known. Studies of drug addiction received an increased impetus when the primary physiological target in the brain was thought to be determined, namely the DA neurons from the VTA innervating the nucleus accumbens. All drugs of abuse share similar neurobiological property, since they increase DA release in the nucleus accumbens that parallels the reinforcing properties of the drugs (Di Chiara and Imperato, 1988; Di Chiara, 1992; Koob, 1992). While the action of drug of abuse on DA neurons can be viewed as an initiating step, the addiction is a much more complex behavioral disturbance. It involves alterations of neurobiological and humoral mechanisms in sequential steps (Le Moal and Simon, 1991; Koob and Le Moal, 1997, 2008; Koob et al., 2014), and is marked by inter-individual differences, spreading drug addiction as a disease (Deroche-Gamonet et al., 2004; Ahmed, 2010). In spite of the numerous studies focusing on the activity of DA neurons as a possibility to avoid relapse and to reduce drug intake, it is possible that DA transmission is no longer an appropriate target once addiction is established. At present, no DA drug is given to drug addicts although some substituted drugs targeting the DAT are developed (Hiranita et al., 2014) by analogy with methadone for opiates abuse.

#### Mechanism of action

The mechanisms of action of drugs of abuse toward DA neuronal activity differ according to the drug of abuse, and most of them, classically, enhance DA outflow through an increase in impulse flow, whereas psychostimulants promote DA outflow independently from DA neuronal impulse. Gaetano Di Chiara's team has first demonstrated that this distinction was probably fundamental, in view of the control of DA neuronal activity and subsequent behavioral outcomes that can be expected from various targets, particularly 5-HT_3_ receptors (Carboni et al., 1989). 5-HT_3_ receptor antagonists counteracted place preference and the enhancement of DA release induced by drugs enhancing DA neuron firing rate. After that discovery (see De Deurwaerdère and Di Giovanni, 2016), 5-HT_2A_ receptor antagonists were shown to counteract the non-exocytotic release of DA induced by amphetamine or 3,4-Methylenedioxymethamphetamine (ecstasy), whereas 5-HT_2C_ receptor agonists inhibited the impulse-dependent release of DA. In parallel, behavioral data have shown that 5-HT_2C_ receptor agonists and 5-HT_2A_ receptor antagonists were capable of dampening the reinforcing properties, the craving and relapse, produced by drugs of abuse, in particular cocaine (Fletcher and Higgins, 2011; Higgins and Fletcher, 2015; Howell and Cunningham, 2015). However, the involvement of DA mechanisms in the benefit of these 5-HT compounds is far from clear, suggesting that the measurement of DA neuronal activity or release is not the most appropriate neurobiological marker of the anti-addictive behavior of therapeutic approaches (De Deurwaerdère and Di Giovanni, 2016).

Other monoaminergic approaches gave promising preclinical results, especially D_3_ receptor antagonists (Leggio et al., 2016). A better determination of the involvement of other systems in addiction might lead to complete new treatment strategies.

## Diseases without current monoaminergic treatments

### Epilepsy

Epilepsy is characterized by seizures whose magnitude varies from almost undetectable to intense shaking. Sudden and unprovoked synchrony of bursts of neuronal hyperactivity can be “focal” if it remains confined to the area of origin, or “generalized.” The origin of this condition is unknown even though genetic predisposition factors have been identified in some cases. Although it manifests as abnormal neuronal activity, it is possible that glial cells play a substantial role in triggering or maintaining this abnormality (Devinsky et al., 2013). Abnormal astrocytic function has been found in models of epilepsy, including the inability to maintain an appropriate extracellular milieu through different mechanisms (Coulter and Steinhäuser, 2015).

Monoaminergic drugs are not in the armamentarium of anti-epileptic drugs, although they have been the object of numerous articles. The monoamine enhancing antidepressant drugs were contraindicated in patients with epilepsy (Jobe and Browning, 2005). Nevertheless, antidepressants such as SNRIs or SSRIs were commonly prescribed to patients to reduce symptoms of comorbid depression and/or anxiety (Cardamone et al., 2013). Against the initial assumption, preclinical and human studies tend to indicate that SSRIs or SNRIs have an anticonvulsant profile, which can improve seizure outcomes (Cardamone et al., 2013). Moreover, SNRIs and SSRIs could limit the occurrence of seizures in depressed patients rather than increase them (Alper et al., 2007). Additional studies are warranted to evaluate the benefit of monoamine-based antidepressant drugs in clinical praxis (see Strac et al., 2016).

SSRIs and SNRIs, as discussed above, can promote numerous mechanisms other than a simple increase in extracellular monoamine levels (Dale et al., 2015). Indeed, one “pseudo-monoaminergic treatment,” namely zonisamide, has received approval for the treatment of epilepsy (Bialer, 2012). Zonisamide interacts with human MAO-B, but not MAO-A (Binda et al., 2011). Similarly, L-deprenyl, the prototypical irreversible MAO-B inhibitor (Ramsay, 2013), has anticonvulsant and antiepileptogenic properties. The mechanisms triggered by these drugs are complex and likely not only related to MAO-B inhibition (Strac et al., 2016).

The roles of individual monoamines and their receptors have been the subject of many studies. To date, no specific target or a combination of specific targets has been approved for epilepsy. In the accompanying paper (Strac et al., 2016), we consider the potential interest of agonistic activity at 5-HT_2C_/D_2_/α_2_-adrenergic receptors and antagonistic activity at H_3_ receptors. It is intriguing that, after years of research, the favorable targets indicated from preclinical studies are those involved in tonic control of neurobiological networks and neuronal activity in the mammalian brain.

### Alzheimer's disease

The oldest of the various hypotheses, proposed in order to explain AD pathogenesis involves the cholinergic system (Sims et al., 1980). Others include the most comprehensive amyloid-β cascade (Karran et al., 2011), glutamate/calcium neurotoxicity (Karran et al., 2011), and hyperphosphorylated tau protein (Li et al., 2007). Recent studies have revealed an association of oxidative imbalance and stress with neuron degeneration (Guidi et al., 2006), and implicated nitric oxide and other reactive nitrogen species (McCann et al., 2005). Accordingly, in addition to the four clinically approved compounds (donepezil, rivastigmine, galantamine, and memantine), that target cholinergic and glutamatergic systems (Parsons et al., 2013), new approaches, focused on the prevention or decline of amyloid cascade events, formation of tangles, oxidative injury, and inflammation, have been proposed for the AD treatment (Ittner and Götz, 2011). It has been suggested that AD patients might benefit from multi-target medication, such as treatment with acetylcholinesterase inhibitors (AChEIs) in combination with memantine (Lopez et al., 2009).

As neuronal degeneration in AD also affects monoaminergic systems (Trillo et al., 2013), dopaminergic and serotonergic dysfunction has been also proposed to be involved (Martorana and Koch, 2014; Claeysen et al., 2015; Šimić et al., 2016), suggesting additional therapeutic targets for AD. Newly synthetized molecules with neuroprotective and multimodal mechanisms of action, currently in different phases of preclinical or clinical investigations (Dias and Viegas, 2014), act not only as AChEI and modulators of Aβ-aggregation (Tonelli et al., 2015), but also at voltage-dependent calcium channels (León and Marco-Contelles, 2011), MAO activity (Bautista-Aguilera et al., 2014), 5-HT_3_ receptors (Fakhfouri et al., 2012) and N-methyl-D-aspartate (NMDA) receptors (Simoni et al., 2012).

Reduced DA levels in the brain (Storga et al., 1996), cerebrospinal fluid (CSF) (Tohgi et al., 1992) and urine (Liu et al., 2011), as well as lower brain levels of L-DOPA and DOPAC have been found in AD patients (Storga et al., 1996). Individuals with AD also exhibit reduced expression of DA receptors (Kumar and Patel, 2007), DAT, and TH (Joyce et al., 1997) in different brain regions. The COMT Val158Met polymorphism, which affects the COMT activity, has been associated with the risk of AD (Yan et al., 2016). The role of dopaminergic system in AD was confirmed by findings showing that L-DOPA (Martorana et al., 2009), the MAO-B inhibitor l-deprenyl (Filip and Kolibás, 1999), and the D_2_ agonist rotigotine (Koch et al., 2014), have beneficial effects on cognitive functions in AD patients. The Aβ accumulation seems to be involved in dopaminergic dysfunction, contributing to the extrapyramidal deficits and rapid cognitive decline in AD patients (Preda et al., 2008). Inhibitors of COMT, like tolcapone, reduce Aβ aggregation by stabilizing its monomeric state (Di Giovanni et al., 2010), while the DA receptor agonist apomorphine promotes Aβ degradation and improves memory (Himeno et al., 2011). While most treatments in clinical trials tend to increase DA function, the antipsychotic drugs haloperidol and risperidone have reached the phase IV of clinical trials (Šimić et al., 2016).

Degeneration of NA neurons, observed in the early stages of AD, in mild cognitive impairment, and even in younger individuals with still normal cognitive function, indicates that alterations in noradrenergic system might precede Aβ deposition (Arai et al., 1992; Grudzien et al., 2007; Braak and Del Tredici, 2011). Lower levels of NA in CSF (Liu et al., 1991), TH in the brain, and DBH in the brain and plasma (Iversen et al., 1983; Mustapic et al., 2013) as well as decreased binding of α_2_ adrenergic receptors (which inhibit the release of NA) in the brain are found in AD patients (Kalaria and Andorn, 1991; Pascual et al., 1992). However, some studies proposed compensatory mechanisms in AD resulting in increased levels of NA (Elrod et al., 1997; Peskind et al., 2005) and TH (Szot et al., 2006).

NA pharmacotherapy has been used primarily to treat different behavioral and psychological symptoms of dementia (BPSD). Propranolol was proposed for the treatment of aggression or agitation (Greendyke et al., 1986; Shankle et al., 1995), and imipramine for treatment of depression in AD (Reifler et al., 1989). However, NA-enhancing therapy could be also used to treat cognitive impairments in patients with dementia. Combination of the NA prodrug L-DOPS and the NET inhibitor atomoxetine was shown to elevate NA levels, increase expression of brain-derived neurotrophic factor and different enzymes involved in Aβ degradation, resulting in improved spatial memory (Kalinin et al., 2012). Reboxetine is currently tested in clinic (Šimić et al., 2016).

Studies suggest that NA deficiency induces inflammatory response by elevating Aβ deposition and reducing microglial Aβ phagocytosis (Heneka et al., 2002, 2010), thus contributing to AD progression. Moreover, it appears that low DBH activity, associated with -1021C/T DBH regulatory polymorphism, increases the risk for AD in combination with interleukin 1 alpha polymorphisms (Mateo et al., 2006; Combarros et al., 2010). These findings suggest the potential of NA to slow down the neurodegeneration process by stimulating anti-inflammatory responses, microglial migration and phagocytosis, thereby contributing to Aβ clearance. Future studies should evaluate proposed anti-inflammatory and neuroprotective mechanisms of NA pharmacotherapy as therapeutic strategy in AD patients.

Lower number/activity of 5-HT neurons (Aletrino et al., 1992; Meltzer et al., 1998), as well as decreased concentration of 5-HT and its metabolites in the brain (Garcia-Alloza et al., 2005), CSF (Tohgi et al., 1992) and blood platelets (Muck-Seler et al., 2009) suggested a role of the serotonergic system in AD (Terry et al., 2008; Ramirez et al., 2014). A progressive decline in the density of different brain 5-HT receptors was observed in AD patients (Reynolds et al., 1995; Lai et al., 2003; Garcia-Alloza et al., 2004; Kepe et al., 2006; Lorke et al., 2006; Marner et al., 2012), sometimes correlating with the severity of cognitive impairment (Versijpt et al., 2003; Lai et al., 2005). Such changes in the serotonergic system could be also associated with BPSD (Lanari et al., 2006).

The association of 5-HT signaling with accumulation of Aβ plaques (Holm et al., 2010; Cirrito et al., 2011), and improvement in cognitive performance after treatment with 5-HT modulators (Payton et al., 2003; Geldenhuys and Van der Schyf, 2011; Ramirez et al., 2014), point to serotonergic system as a potential target for AD therapy. Both *in vivo* and *in vitro* studies demonstrated the potential of different agonists and/or antagonists of various 5-HT receptors for treating AD, including those affecting 5-HT_1A_, 5-HT_4_, and 5-HT_6_ receptors (Meneses, 2013; Ramirez et al., 2014; Šimić et al., 2016; Werner and Covenas, 2016). The development of serotonergic medications which interact with multiple targets (Rezvani et al., 2009; Fakhfouri et al., 2015; Leiser et al., 2015; Stahl, 2015), could be a promising approach for AD treatment. The most advanced clinical trials focus on the 5-HT_1A_ receptor agonists, tandospirone or buspirone, as well as citalopram (Šimić et al., 2016).

Multitarget drug design is also used, targeting enzymes involved in oxidative stress and inflammation, and these strategies are likely to interfere with monoamine tone. This research is presented in the next section.

### Ischemic stroke/oxidative stress

Stroke is a vascular disorder that constitutes the second leading cause of death worldwide with higher incidence in elderly people. Inflammation and oxidative stress, accumulated during human aging, negatively influence the vascular damage following a stroke incident (DiNapoli et al., 2008), and the immune system may contribute to the infarct progression (Iadecola and Anrather, 2011). Human brain is highly demanding of oxygen. It accounts for the 20% of the body oxygen consumption. This high demand of energy makes the brain highly dependent on cerebral blood supply and an organ especially susceptible to the deleterious effect of hypoxia. Two different types of stroke occur. Ischemic stroke that occurs in the blood vessels of the brain as a consequence of the formed clots, preventing oxygen arrival to the brain cells and inducing a hypoxic condition. About 80% of all strokes are ischemic. On the other hand, the hemorrhagic stroke occurs when a blood vessel in the brain breaks or ruptures as a consequence of high blood pressure or brain aneurysms, resulting in the blood seeping into the cerebral tissue, causing damage to the brain cells. Actually, the fact that a high percentage of patients having suffered stroke subsequently developed AD, indicates a strong link between these two pathologies. In this context, hypoxia and ischemic injury induce the up-regulation of beta secretase 1 (BACE-1) that increases the β-amyloid formation (Guglielmotto et al., 2009). Increasing evidence suggest that the neurovasculature plays an important role in the onset and progression of neurological disorders like AD (Zlokovic, 2008; Grammas, 2011; Marchesi, 2014). This has led to the concept of “neurovascular unit,” integrated by neurons, astrocytes and vascular cells, which constitutes a functional unit able to maintain the homeostasis of the brain's microenvironment (Iadecola, 2010). Different amine oxidases are present in this “neurovascular unit” and they could play an important role under physiological and pathological conditions.

As discussed above, MAOs are involved in the metabolism of biogenic amines. MAO-Is provide additional benefits in neurologic disorders by reducing the formation of neurotoxic products such as hydrogen peroxide and aldehydes, which are derived from MAO activities. In neurodegeneration, MAO-I, especially those containing a propargylamine group, have been reported to possess multiple beneficial activities including neuroprotective, anti-apoptotic and antioxidant properties (Jenner, 2004; Sanz et al., 2004, 2008; Bar-Am et al., 2005).

Another amine oxidase present in cerebrovascular tissue, mainly in endothelial cells constituting the BBB, also plays a role in neurological disorders. Vascular adhesion protein 1 (VAP-1) is a homodimer glycoprotein with enzymatic function that binds leukocytes through its semicarbazide sensitive amine oxidase (SSAO) activity inducing inflammation (Smith et al., 1998; Jalkanen and Salmi, 2008). SSAO/VAP-1 metabolizes only primary amines producing aldehydes, hydrogen peroxide and ammonia, which are able to induce oxidative stress and cellular damage when overproduced (Yu and Deng, 1998). SSAO/VAP-1 is localized at the cell membrane and is released into blood as soluble form that is altered in several human pathologies (Kurkijärvi et al., 1998; Boomsma et al., 2003), including AD (Ferrer et al., 2002; del Mar Hernandez et al., 2005) and cerebral ischemia (Airas et al., 2008). The mediators that induce these alterations in the SSAO/VAP-1 levels are still unknown, but it is believed that increased SSAO/VAP-1 levels may contribute to the physiopathology of these diseases, thus constituting a potential therapeutic target (Conklin et al., 1998; Solé et al., 2008). Actually, human plasma SSAO activity is a strong predictor of parenchymal hemorrhages after tissue plasminogen activator (tPA) treatment in ischemic stroke patients (Hernandez-Guillamon et al., 2010), and plasma SSAO activity, which is also elevated in hemorrhagic stroke patients, predicts their neurological outcome (Hernandez-Guillamon et al., 2012). Considering all these data, SSAO/VAP-1 could mediate the link between stroke and the progression to AD through the alteration of the cerebrovascular function.

In spite of the different cellular localization of both MAO isoforms as well as SSAO/VAP-1, and their different substrate specificities, a cross-talk between these oxidases occurs. It has been described that MAO modulates the behavior of SSAO/VAP-1 (Fitzgerald and Tipton, 2002) and SSAO/VAP-1 may compensate in some extent the deficit in the oxidative deaminating capacity in situations where MAO activity is dysfunctional (Murphy et al., 1991).

Dynamic communication between cells of the neurovascular unit is required for normal brain functioning. The neurovascular unit consists of all major cellular components of the brain including neurons, astrocytes, brain endothelium, pericytes, vascular smooth muscle cells, microglia, and perivascular macrophages. In a situation of hemorrhagic stroke, an alteration of the homeostasis between all components of the neurovascular unit occurs, and the role of amine oxidases, especially the SSAO/VAP-1 activity is increased, contributing through its catalytic activity to the oxidative stress and inflammation (Hernandez-Guillamon et al., 2012). The neurovascular unit disruption may also alter the neuronal monoaminergic transmission. In this context, a pharmacological approach that is able to interact simultaneously with different amine oxidases might be a novel and more effective therapeutic approach (Solé et al., 2015; Sun et al., 2015). The close relationship between AD and stroke and the involvement of SSAO/VAP-1 in both diseases suggests that the synthesis and design of the new multitarget drugs, able to interact with different types of amine oxidases (MAO-A, MAO-B, and SSAO/VAP-1), could provide a useful therapeutic approach that is able to modulate the monoaminergic transmission, to protect the BBB, and hence avoid the progression of both neurological disorders. It should be noted that oxidative stress is recurring feature of several theories of the etiology of neurodegenerative diseases, which might be addressed by reducing hydrogen peroxide formation.

### Concluding remarks

Despite the progress made on the influence of monoamines in the three above-mentioned diseases, no monoamine-based treatments are available at present. In most cases, one emerging strategy would be the use of SSRI or MAO-Is, two approaches that are altering monoaminergic transmission throughout the brain rather than a specific monoaminergic drug targeting the injured tissue. In fact, there are two interconnecting issues: one addressing the regional origin of the disease, and the other addressing the functional consequences away from the dysfunctional site. Assuming that the benefit of antidepressant drugs like SSRI or MAO-I is related to the ultimate reorganization of neurobiological networks, their possible benefit could be related to actions away from the injured sites.

## General concluding remarks

Monoaminergic drugs affect different processes in the brain that control the tone of neurotransmission or/and tissue excitability, and therefore should be studied as future targets for novel therapeutic approaches, not only for depression, but also for schizophrenia, addiction, obesity, PD, epilepsy, AD and stroke. This analysis acknowledges the Neuroscience based Nomenclature proposed by the IUPHAR which is based on the pharmacological profiles of psychotropic drugs rather than the clinical indication (Zohar et al., 2015). Common approaches involve the use of agents acting on monoaminergic transporters, D_2_, D_3_, 5-HT_2B/2C_, 5-HT_1A_, 5-HT_2A_, H_3_, or α_2_ receptors. All these targets, apart the 5-HT_2A_ receptor, exert widespread influences in the brain and alter multiple brain functions simultaneously. Thus, compounds affecting monoaminergic transmission have numerous consequences for the brain and its neurobiological networks. While researchers are aware that the symptomatic treatment of a disease results from multiple actions on the brain function, the linkages enabling a favorable reorganization of brain function after monoamine-based treatments are still not fully understood.

The search for effective targets in the treatment of brain diseases was facilitated by the serendipitous discoveries of compounds that were efficacious in schizophrenia, depression and obesity. Several new compounds have been developed that retain the original pharmacological targets involved in those therapeutic benefits combined with actions on new targets, identified from subsequent research, whilst aiming to minimize undesirable “off-target” actions. Knowledge of the monoaminergic mechanisms underlying drug efficacy led to the development of drugs with more specific mechanisms of action; lorcaserin, agomelatine, and vortioxetine are interesting achievements in this context. The identification of pathophysiological causes to tailor appropriate therapeutic approaches has been less successful. It has shown some success with PD but not with addiction to drug of abuse. Nonetheless, the failure to improve on current antiparkinsonian therapies confirms that the efficacy of these treatments is not simply related to a compensation of the loss of striatal DA. To some extent, the remarkable clinical success of L-DOPA was serendipitous. It is difficult to implement new monoaminergic treatment strategies in the clinic unless they depend on those currently accepted. This is exemplified by the third group of diseases where the progress regarding the involvement of monoamines in these diseases has not yet resulted in new monoamine-based treatments.

Current attempts to address the full spectrum of action of existing drugs can be improved. It is to be hoped that novel approaches, such as connectomics with polyomic research addressing brain functions, neurobiological networks, monoaminergic systems and their mutual interactions, might offer new perspectives and enhance the current state-of-the-art biological research in these psychiatric and neurological disorders.

## Author contributions

All authors contributed to the conception and interpretation of the work and to its critical revision. All authors have approved the final version and may be held accountable for the integrity of this review of current literature.

### Conflict of interest statement

The authors declare that the research was conducted in the absence of any commercial or financial relationships that could be construed as a potential conflict of interest.

## Abbreviations

COMT: catechol-O-methyl transferase
DA: dopamine
5-HT: serotonin
NA: noradrenaline
MAO: monoamine oxidase
MAO-I: monoamine oxidase inhibitor
SSRI: selective serotonin reuptake inhibitor
SNRI: serotonin noradrenaline reuptake inhibitor
AD: Alzheimer's disease
PD: Parkinson's disease
VTA: ventral tegmental area
SNc: substantia nigra pars compacta
LC: locus coeruleus
L-DOPA: L-3,4-dihydroxyphenylalanine
AADC: aromatic L-amino acid decarboxylase
DBH: dopamine β-hydroxylase
TPH: tryptophan hydroxylase
VMAT2: vesicular monoamine transporter
SERT: 5-HT transporter
DAT: DA transporter
NET: NA transporter
OCT: organic cation transporters
PMAT: plasma membrane monoamine transporter
EPS: extrapyramidal side-effects
TCA: tricyclic antidepressant
AChEI: acetylcholinesterase inhibitor
VAP-1: vascular adhesion protein 1.
